# Interfacial Stress in the Development of Biologics: Fundamental Understanding, Current Practice, and Future Perspective

**DOI:** 10.1208/s12248-019-0312-3

**Published:** 2019-03-26

**Authors:** Jinjiang Li, Mary E. Krause, Xiaodong Chen, Yuan Cheng, Weiguo Dai, John J. Hill, Min Huang, Susan Jordan, Daniel LaCasse, Linda Narhi, Evgenyi Shalaev, Ian C. Shieh, Justin C. Thomas, Raymond Tu, Songyan Zheng, Lily Zhu

**Affiliations:** 1 Pharmaceutical Development, Wolfe Labs, 19 Presidential Way, Woburn, Massachusetts 01801 USA; 2 Drug Product Science and Technology, Bristol-Myers Squibb Company, One Squibb Drive, New Brunswick, New Jersey 08901 USA; 3 Formulation Development, Regeneron Pharmaceuticals, Inc., Tarrytown, New York 10591 USA; 4 Large Molecule Drug Product Development, Janssen Research & Development, LLC, Johnson and Johnson, Malvern, Pennsylvania 19355 USA; 5 BioProcess Technology Consultants, Woburn, Massachusetts 01801 USA; 6 Department of Bioengineering, University of Washington, Seattle, Washington 98195 USA; 7 Biotherapeutics Pharmaceutical Sciences, Pfizer, Andover, Massachusetts 01810 USA; 8 Pharma Excipients, The Dow Chemical Company, Collegeville, Pennsylvania 19426 USA; 9 Process Development, Amgen, Inc., Thousand Oaks, California 91362 USA; 10 Pharmaceutical Development, Allergan Inc., Irvine, California 92612 USA; 11 Late Stage Pharmaceutical Development, Genentech, Inc., South San Francisco, California 94080 USA; 12 Bioproduct Research & Development, Eli Lilly and Company, Indianapolis, Indiana 46285 USA; 13 Department of Chemical Engineering, The City College of New York—CUNY, New York, New York 10031 USA; 14 Technical Operations, CRISPR Therapeutics, Cambridge, Massachusetts 02139 USA

**Keywords:** aggregation, analytical methods, biotherapeutic, interfacial stress, product development, Protein

## Abstract

Biologic products encounter various types of interfacial stress during development, manufacturing, and clinical administration. When proteins come in contact with vapor–liquid, solid–liquid, and liquid–liquid surfaces, these interfaces can significantly impact the protein drug product quality attributes, including formation of visible particles, subvisible particles, or soluble aggregates, or changes in target protein concentration due to adsorption of the molecule to various interfaces. Protein aggregation at interfaces is often accompanied by changes in conformation, as proteins modify their higher order structure in response to interfacial stresses such as hydrophobicity, charge, and mechanical stress. Formation of aggregates may elicit immunogenicity concerns; therefore, it is important to minimize opportunities for aggregation by performing a systematic evaluation of interfacial stress throughout the product development cycle and to develop appropriate mitigation strategies. The purpose of this white paper is to provide an understanding of protein interfacial stability, explore methods to understand interfacial behavior of proteins, then describe current industry approaches to address interfacial stability concerns. Specifically, we will discuss interfacial stresses to which proteins are exposed from drug substance manufacture through clinical administration, as well as the analytical techniques used to evaluate the resulting impact on the stability of the protein. A high-level mechanistic understanding of the relationship between interfacial stress and aggregation will be introduced, as well as some novel techniques for measuring and better understanding the interfacial behavior of proteins. Finally, some best practices in the evaluation and minimization of interfacial stress will be recommended.

## INTRODUCTION

Protein therapeutics encounter various types of interfacial stress during development, manufacturing, and clinical administration. When proteins come in contact with vapor–liquid, solid–liquid, and liquid–liquid surfaces, these interfaces can significantly impact the protein drug product quality attributes, including formation of visible particles, subvisible particles, or soluble aggregates, or changes in target protein concentration due to adsorption of the molecule to various interfaces. Protein aggregation at interfaces is often accompanied by changes in conformation, as proteins modify their higher order structure in response to interfacial stresses such as hydrophobicity, charge, and mechanical stress. Formation of aggregates may elicit immunogenicity concerns; therefore, it is important to minimize opportunities for aggregation by performing a systematic evaluation of interfacial stress throughout the product development cycle and to develop appropriate mitigation strategies.

Various types of interfacial stress are encountered throughout both drug substance and drug product processing, storage, and clinical use. The protein isolation and purification steps used in the drug substance manufacturing process involve interaction of the molecule with various solid/liquid interfaces, such as columns and filters, processing equipment, container surfaces, and ice during storage. The molecule is also exposed to air/liquid interfaces as part of mixing and mechanical pumping operations. During drug product manufacturing, the proteins are thawed and often mixed with additional excipients before being filled into vials or syringes. The thawing process exposes the protein to the ice/liquid interface, then the mixing step exposes the protein to the air/water interface while being sheared, before the filling process exposes the molecule to high shear for a short period of time. Once filled, opportunities for air/liquid interfacial stress are the primary packaging, the container’s headspace, agitation during transport, and stress during clinical administration. Administration of the drug product is achieved using either IV bags, syringes, or autoinjectors, in which the protein is exposed to a variety of surfaces and materials, including plastics of IV bags and infusion sets, in-line filters, silicone oil, and metals.

The aims of this white paper are to provide an understanding of protein interfacial stability, explore methods to understand interfacial behavior of proteins, and then describe current industry approaches to address interfacial stability concerns. Specifically, we will discuss interfacial stresses to which proteins are exposed from drug substance manufacture through clinical administration, as well as the analytical techniques used to evaluate the resulting impact on the stability of the protein. An overview of the mechanistic understanding of the relationship between interfacial stress and aggregation will be introduced, as well as some novel techniques for measuring and better understanding the interfacial behavior of proteins. Finally, some best practices in the evaluation and minimization of interfacial stress will be recommended.

## CORRELATION BETWEEN INTERFACIAL STRESS AND AGGREGATION

**Fig. 1:**
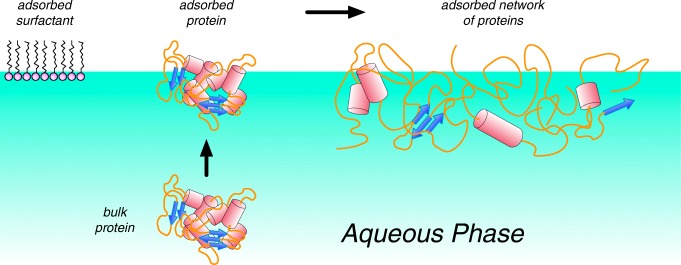
Protein interfacial behavior. Proteins from the bulk solution can adsorb to the interface leading to an adsorbed network of proteins. Surfactants can mitigate this adsorption. Modified from Morris *et al.* (1)

Aggregation of therapeutic proteins can be induced by stresses encountered at vapor–liquid, solid–liquid, and liquid–liquid interfaces (Fig. 1) (1). Aggregation typically involves some degree of protein conformational change, relative to the folded monomer, that allows two or more proteins to form interprotein bonds that possess similar or greater stability than the intraprotein bonds of the native folded structure. The corresponding growth of aggregates typically occurs through addition of monomers and/or through combining aggregates to form soluble high molecular weight (HMW) aggregates. The mechanism by which the stresses encountered at interfaces promote protein aggregation relative to that in the bulk of solution is less well established. Whether the proteins distort/unfold and self-assemble into aggregates at the interface, and/or whether they release from the interface and further aggregate while in the bulk solution (or even disassemble and refold) is not definitively settled. In principle, each type of interfacial species can reversibly exchange with the corresponding bulk species through convective and/or diffusive mass transport between the bulk and interfaces. The adsorption and desorption processes may be at equilibrium or under mass-transfer control. If under equilibrium control, gentle sample mixing processes are not expected to affect the concentration of adsorbed species; however, if under mass transfer control, then mixing should enhance the adsorption and/or desorption rates *via* convective mass transfer.

While long-term exposure to interfaces can be detrimental to the physical stability of therapeutic proteins, transient exposure to interfaces in combination with mechanical disruption of the interface (*e.g.*, agitation, mixing, pumping) is particularly conducive to causing protein aggregation. Interfacial exposure and shear stress typically occur together, but it is unlikely that the shear stress alone causes protein aggregation (2). Numerous reports in the literature support the hypothesis that protein adsorption to interfaces, followed by subsequent denaturation and aggregation at the interface, leads to formation of protein particulates and soluble aggregates. In particular, due to the dilution of surfactants typically included in formulations to stabilize proteins against interfacial stress, handling and transportation of protein drugs prepared in IV infusion bags can result in substantial aggregation due to the air–liquid interface present in the bags. By removing the air headspace, and thus eliminating the air–liquid interface in the IV bag, the bags can undergo agitation with essentially no protein aggregation (3,4). One marketed biologic, LUMIZYME® (Genzyme Corporation, Cambridge, MA), explicitly instructs for the removal of air from the IV bag “to minimize particle formation because of the sensitivity of LUMIZYME® to air–liquid interfaces” (5). Similar to IV bag agitation, agitation of a mAb in a drug product vial with air headspace led to extensive aggregation, whereas shaking under identical conditions without the air headspace substantially limited the observed aggregation (6). Other investigators have studied the effect of agitation in siliconized *versus* unsiliconized prefilled syringes and found that the presence of silicone oil exacerbated the agitation-induced aggregation of a variety of protein therapeutics (7–9). Because protein adsorption to interfaces is frequently limited to the formation of a monolayer (10), rather than multilayers, increases in protein concentration in the bulk solution can result in an inverse relationship between the percent of protein aggregated *versus* the bulk concentration (11).

Typically, interfaces lead to extensive protein aggregation in combination with a mechanical stress, which deforms, eliminates, or physically perturbs the interfacial protein film. The IV bag agitation study referenced above demonstrated that static storage in the IV bag, even with the air headspace present, did not result in aggregation (3). The formation of protein aggregates during agitation of siliconized prefilled syringes stopped once the agitation halted (7). Similarly for a mAb, halting agitation of a drug product vial also stopped aggregation (12). A fundamental study of mAb aggregation at air–liquid interfaces determined that interfacial shear stress caused substantially more protein particulate formation compared to bulk shear stress. This study also indicated that dilatational compression of antibody films that have been exposed to air for longer, and are therefore more denatured, resulted in shedding of protein particulates into the bulk solution (13). Similarly, repeated lateral compression and expansion of mAb adsorbed to an air–liquid interface resulted in comparable protein aggregation compared to an agitation study (14). While these previous two studies demonstrated compression of interfacial protein films causes protein particulates, other studies showed a similar result for protein films at air–liquid (15) and oil–liquid (16) interfaces only when the films were repeatedly perturbed with a needle during vial rotation. The literature reports referenced here, along with many others, demonstrate that protein therapeutics can aggregate when exposed to interfaces, particularly in combination with mechanical stresses that deform or perturb the interfacial protein layer.

## INTERFACIAL STRESS IN DRUG SUBSTANCE DEVELOPMENT

**Fig. 2:**
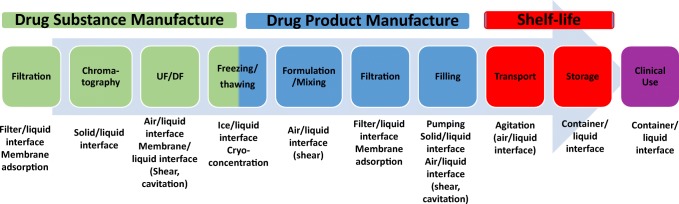
Process flow diagram that indicates types of interfacial stress that can occur during unit operations for drug substance and drug product manufacture, as well as upon transportation and storage

Biologics are developed and manufactured in two major stages. Biologic drug substance is the purified bulk material that has been concentrated to the target protein concentration, partially (or fully) formulated, and typically frozen until initiation of drug product manufacturing. The drug product is the fully formulated protein solution contained in a vial, syringe, or other delivery device that is ready for patient administration. Both protein drug substance and drug product process development and manufacturing workflows can expose proteins to various interfacial stress conditions (Fig. 2). During drug substance manufacturing, the protein is taken through a series of unit operations, including harvest, centrifugation or filtration for removal of cell debris, purification *via* column chromatography, filtration, virus reduction, concentration, and formulation/storage. In particular, the drug substance filtration, freezing/thawing, as well as unit operations that combine mechanical and interfacial stresses are particularly impactful and will be detailed here.

### Filtration

Several steps in the drug substance manufacturing process can lead to interfacial stress due to interaction with various types of filters. First, following protein expression in the production bioreactor, a filtration operation is employed to remove cells and/or solid cellular debris. The industry standard for large-scale manufacturing involves a disk-stack centrifugation step, followed by normal flow depth filtration. Further purification is typically achieved through one or more chromatography operations to isolate the protein of interest, while removing impurities and increasing concentration. Purification of most mammalian cell–derived biologics includes a virus removal step, which is typically a normal flow operation. Additionally, multiple normal flow filters are placed throughout drug substance processing; in particular, particle reduction or microbial control filters are commonly placed before the load and pools of each process step (17–20).

During drug substance manufacturing, protein solutions are often concentrated and/or buffer exchanged using an ultrafiltration/diafiltration (UF/DF) process, which is a membrane-based tangential flow filtration (TFF) process where products are exposed to multiple pump passes, recirculation, and mixing. Likewise, in aseptic drug product manufacturing, compounded protein bulk solutions are filtered for bioburden reduction or sterile filtration using normal flow filtration. While both operations are membrane-based filtration unit operations, the differences are mainly the direction of the fluid flow relative to the membrane and the total time spent in contact with the membrane, which thus affects the total amount of shear and surface exposure the molecule is exposed to throughout the filtration process.

During each of these filtration processes, high levels of shear stress are often generated at the interface of the filter membrane and the protein solution. Interfacial stress may promote protein–protein interactions, or even lead to protein conformational rearrangement to reduce the energy of interaction, which could further lead to protein aggregation or particle formation (21–23). In the ultrafiltration process, the protein concentration at the membrane interface may be much higher than the overall bulk solution. This might further result in membrane fouling and boundary layer polarization near the filter membrane. All these factors could potentially lead to protein aggregation (24–26). The filtration force is closely related to the membrane pore size and the distribution. It has been reported that filtration forces through polyvinylidene fluoride (PVDF) filters are lower than those through polyethersulfone (PES) filters (27). Although both sterile filters have a nominal size of 0.2 μm, scanning electron microscopy (SEM) images collected as part of the study by Allmendinger *et al.* showed that the PVDF filter investigated had larger pore sizes as compared to the PES filter tested. Mercury porosimetry also confirmed that the PVDF filter had a wider pore size and relatively more heterogeneous pore size distribution (27).

In addition to pore size and distribution, the interfacial stress during filtration is also impacted by the surface properties of the filter membrane and the physical properties of the filtrates. It has been reported that the interfacial adsorption–desorption of the protein to and from hydrophobic interfaces during ultrafiltration/diafiltration is the principal cause of particle formation, and the level of surfactant-free particle formation depends on the colloidal stability of the protein (28). Though formed particles can likely be removed through further downstream filtration processes, the exposure of the molecule to this interfacial stress may still potentially have an impact on the filterability of the protein solution. These systems may require alternate strategies, such as performing UF/DF at low conductivity, adding additional stabilizer(s) to improve colloidal stability, or adding surfactants, such as polysorbate, to the process stream (28).

Frequently, polysorbate is found to be a critical excipient that helps to protect the protein solution from interfacial stress during various unit operations (29). However, if included in the UF/DF operation, polysorbates can be retained with the protein. Thus, in many cases, surfactant is not added until after the UF/DF unit operation, so this protectant is not present until the end of drug substance manufacturing. Additionally, because polysorbate adsorption to the membrane is often encountered during the filtration process, any polysorbate adsorption during filtration needs to be carefully evaluated. If significant adsorption is observed, mitigation strategies need to be in place in order to both monitor and control the adsorption, to ensure that the surfactant concentration postfiltration meets the intended requirements. During filtration process development for both drug substance and drug product, laboratory-scale studies are recommended in order to screen suitable filter membranes and choose the right filter with appropriate size. In particular, in certain cases, an appropriate product flush volume may be warranted during drug product manufacture to ensure filter adsorption does not lead to vials with reduced levels of protein or excipients. The predetermined filter flush volume can be assessed based on the filter adsorption behavior in laboratory-scale studies, then further confirmed or adjusted in at-scale studies based on the sterile filtration and filling process during drug product manufacturing.

### Mechanical Shear Stress/Mixing

Mechanical shear stresses may be introduced from a variety of the manufacturing equipment utilized throughout drug substance and drug product manufacturing, including gas sparging, centrifuges, pumps, agitators, and flow-through plumbing. Generally, highly polished, slower moving mechanical parts are designed into the process equipment, and where required, pumps with lower RPM and low shear impellers are employed. Plumbing and piping are typically sized to ensure linear velocities remain low enough to avoid highly turbulent flow regimes, typically targeting < 5 ft/s (19). To assess the susceptibility of a biologic to mechanical shear degradation, lab-scale risk assessment tools such as flow-through capillaries or rotating disks have been employed (30,31).

Across the drug substance manufacturing process, air/liquid interfaces must be considered and minimized to prevent negative impacts to the protein of interest. Due to sparging in bioreactors, shear protectants are employed to prevent damage to the cells (32,33), while antifoam agents are employed to prevent foaming (34). Plumbing and pumps are designed to operate fully flooded to prevent unnecessary exposure to air/liquid interfaces. Process-intermediate mixing should be controlled to maintain gentle agitation and avoid the formation of a vortex, while ensuring agitators remain fully submerged. Stainless steel tanks are commonly used for process pool collection or TFF retentate recirculation, where solution flows through a dip tube into the tank. The design and location of these dip tubes are important to minimize and avoid violent air/liquid interfaces and prevent foaming. To assess the susceptibility of a biologic to air/liquid interfaces, a variety of methods have been employed, including shaking in a partially filled vial, mixing with deep vortex, and mixing with partially submerged agitator.

Several mixing unit operations take place during both drug substance and drug product manufacturing processes, such as diluent mixing and concentrate preparation; adjusting the solution pH, concentration, and/or conductivity to the appropriate conditions for the subsequent process step; *etc*. These mixing operations are often performed in mechanically agitated tanks. During the agitation step, inappropriate mixing conditions may lead to extensive foaming and/or cavitation for protein solutions. The air/liquid interface is hydrophobic, resulting in the adsorption of surface-active molecules like proteins to this surface. The interfacial exposure may lead to at least partial unfolding of the protein, hence exposing their hydrophobic core and promoting protein–protein interactions and aggregation. Stirring forces these partially unfolded species into the bulk and allows more protein to be exposed to the interface. For instance, recombinant human growth hormone solutions (0.5 mg/mL) mixed by vortexing for just 1 min have been shown to lead to as much as 67% of the protein precipitating as noncovalent insoluble aggregates (35).

A recent review paper from Thomas *et al.* focuses on the effects of shear stress on proteins in solution (2). This work suggests that protein aggregation or instability is not solely induced by mechanical shear, and other factors such as interfacial effects are often found to be the primary mechanisms, or at least significant contributing factors. Kiese *et al.* evaluated the impact of agitation on proteins by using different agitation methods and found that shaking and stirring resulted in the formation of different numbers of particles (36).

Appropriate mixing parameters are critical to ensure a homogeneous product while minimizing shear-induced product degradation throughout the manufacturing process. Characterization of mixing processes, determination of the overall level of mixing, and understanding the behavior of the protein in the mixing tank are all critical for manufacturing, especially during the compounding process. In particular, bottom-mounted mixers can impart significant shear stress on the bulk drug product during mixing operations (37). Generally, stirred mixing tanks can be evaluated through experimental investigation of the impeller design, tank geometries, and fluid rheology; however, due to material limitations during the earlier development stages of drug product manufacturing, the at-scale experimental approach could be challenging. Instead, platform knowledge of the mixing unit operation can be leveraged, or scaled-down experiments can be performed. The pH, osmolality, and/or protein concentration can be measured through sampling from the bottom, middle, and top of the mixing tank. Additionally, protein solutions should be exposed to various interfacial and shear stresses, with subsequent analysis of specific critical and product quality attributes (for example, size variants such as formation of particulates, subvisible particles, and soluble aggregates; chemical modification of the monomer; *etc*.) (38). Establishment of laboratory scale down models to qualify the design space for the process parameters of these key unit operations by simulating the normal operation ranges (NOR) as well as proven acceptable ranges (PAR) for unit operations is recommended. As an alternative to scale down models, an at-scale worst-case mixing study could be performed as part of engineering runs or performance qualification runs.

### Impact of Freeze–Thaw on Protein Stability

Biologic drug substances are typically frozen for long-term storage, and then thawed at the start of drug product manufacture. Drug product containers can also experience unintentional frozen excursions during shipping and/or storage. While a frozen drug substance allows time to elapse before drug product manufacture without affecting overall stability, and permits the manufacture of multiple drug products from a single drug substance lot, multiple freeze/thaw cycles can have a negative impact on product quality. Multiple stresses are associated with freeze–thaw, including freeze concentration, pH changes, mechanical stresses, and low temperature per se which could result in cold denaturation. In addition, freezing (water-to-ice transformation) creates extensive ice/solution interfaces, and it has been suggested that freeze-induced destabilization of proteins is related to exposure of the protein molecules to these interfaces (39). As formulated proteins freeze, the formation of ice crystals excludes the solutes and protein, causing cryoconcentration. In formulations that contain a crystallizable solute, such as NaCl, mannitol, PEG, sorbitol, or trehalose, freezing can result in secondary solute+water crystallization (40–43). The freeze-concentrated solutions (FCS) can then be exposed to either these crystals at the resulting solute/FCS interface, or to ice/FCS interfaces.

A second type of freezing-created interface that can lead to protein destabilization is caused by the formation of air bubbles during freezing. As a material is frozen, growth of ice crystals results in increase in the concentration of all solutes present in the sample, including dissolved air gasses (predominantly nitrogen and oxygen). As these gasses become more concentrated, air bubbles form, creating additional pathways for surface-related protein instability due to newly formed solution/air interface (44). The appearance of air bubbles during freezing has been directly observed by optical microscopy (45) and indirectly by small-angle neutron scattering (SANS) (46). Protein adsorption onto these freezing-formed air bubbles and protein destabilization due to the additional air/solution interface can therefore be expected, although the relative significance of this potential destabilization mechanism has not been studied. Finally, additional interfaces could also be created in the maximally freeze-concentrated solution when it is cooled below the glass transition temperature, as suggested in a recent study of a model sorbitol–water system (47). A sorbitol–water mixture with a composition representative of the maximally freeze-concentrated solution was studied by the SANS method. It was reported that cooling of the sample below its calorimetric Tg (glass transition; approx. 210 K) resulted in the development of domains with well-defined and sharp interfaces on the submicrometer length scale, probably as a result of the appearance and growth of microscopic voids in the glassy matrix. Importantly, these interfaces persisted even when the sample was heated back to above its Tg; therefore, these interfaces could represent an additional source of protein surface-related instability during freeze–thaw and freeze–drying/reconstitution.

Several key studies have demonstrated clear correlations between freezing (*i.e.*, ice formation) and protein instability. Conformational changes of globular proteins were studied by employing the phosphorescence emission of tryptophan (Trp) residues as a monitor of the conformational changes of six proteins in response to variations in conditions of the medium (48). Changes in well-structured compact cores of the macromolecules were monitored by the direct correlation between the phosphorescence lifetime *τ* and the rigidity of the protein matrix surrounding the chromophoric probe. The solidification of water markedly decreased *τ* and indicated unfolding-related changes in conformation of the proteins, which was related primarily to the protein–ice interaction. Additionally, Trp florescence was employed to monitor unfolding of an azurin mutant C112S from *Pseudomonas aeruginosa*. The results obtained with C112S azurin indicated that the stability of the native fold may be significantly perturbed in the frozen solutions depending mainly on the size of the liquid solution pool in equilibrium with the solid phase (49).

In more recent studies, evidence of partially unfolded proteins at ice interfaces has been observed by infrared microscopy (39,50,51). We should note that, while these studies provided convincing evidence that destabilization of protein could be induced by formation of ice, it is not obvious if such destabilization is the result of the direct protein/ice interaction, as other mechanisms can be invoked. For example, it was suggested that the observations of freeze-induced protein destabilization do not necessarily prove that there is a direct ice/protein interaction (*i.e.*, sorption of protein molecules on ice crystals), as the same results could be explained by a partitioning of protein into quasi-liquid layer (52). The quasi-liquid layer (also known as liquid-like layer) is a thin film of liquid water on the surface of ice crystals, which exists well below the ice melting temperature even in pure water (53). Estimations of the thickness of the layer vary widely, from a few angstroms to up to a micrometer (54). Protein molecules in the quasi-liquid layer could be exposed to destabilizing stresses, such as an increased local acidity due to the negative charge on the surface of ice crystals (55). The ice surface was shown to carry negative charge when in equilibrium with solution at pH values higher than 4; the negative charge is balanced by the elevation in the local concentration of cations, including protons, in the quasi-liquid layer next to the ice surface, with the corresponding increase in the local acidity.

Understanding the mechanism for freeze-induced protein stabilization is essential to the development of appropriate stabilization strategies. For example, if a main destabilization pathway were direct sorption of protein molecules onto ice, minimization of surface area of ice crystals would be a sensible approach. On the other hand, if protein is partitioned in the quasi-liquid layer or resides predominantly in the bulk freeze-concentrate, stabilization would involve modification of the amorphous protein environment, where the following approaches could be considered:

- Minimize partitioning of the protein into the layer (*e.g.*, more time for protein to diffuse out of the layer to the bulk)
- Reduce acidity gradients between the surface of ice crystals, quasi-liquid layer, and the bulk (*e.g.*, increase salt concentration)
- Reduce pressure built-up (*e.g.*, slower growth of ice crystals)
- Avoid phase separation by use of a cryoprotector

To conclude, evaluation of freeze–thaw stability is a necessary part of any protein formulation development process (56). It was also pointed out that freeze–thaw cycling experiments, which are usually performed with smaller samples sizes, are not necessary predictive of long-term frozen stability of proteins in larger amounts (57). Stability differences between small- and large-scale samples of the same formulation could be related to different ice nucleation and crystal growth patterns and, therefore, variations in interfaces proteins are exposed to on different scales. Accordingly, a surfactant such as polysorbate, which competes with proteins for interfaces, can improve the stability of frozen proteins, as demonstrated, *e.g.*, by Schwegman *et al.* (50).

## INTERFACIAL STRESS IN DRUG PRODUCT DEVELOPMENT

During drug product manufacturing, a drug substance may be mixed with additional excipients, often after thawing, before being filled into vials, syringes, and other devices. The thawing process exposes the protein to the ice/liquid interface, then the mixing step exposes the protein to the air/water interface while being sheared *via* stirring. The effects of these stresses on biologics were described above. However, the unit operation that likely results in the most interfacial stress is the filling process. Once filled into vials, opportunities for exposure to interfacial stress emerge during storage, transport, and clinical administration.

### Drug Product Filling

The drug product filling process can affect product quality attributes, since the protein solution may be exposed to shear, friction, and cavitation during this unit operation. Several factors, including the filling pump mechanism, contact material, filling speed, and filling needle size, can all affect the overall stress encountered by a protein during the filling operation. For instance, Noyak *et al.* reported that the rotary piston pump generated more protein subvisible particles compared to the rolling diaphragm pump, peristaltic pump, and time-pressure pump (58). Any foreign particles generated during filling will not only affect protein stability but also pose a significant risk of causing immunogenicity (59,60). The authors attributed possible factors to be the narrow annular gap that produced high shear and friction, as well as the maximum exposure of the product to metallic/ceramic surfaces during filling. Biddlecombe *et al.* (61) have also reported significant levels of protein aggregation and precipitation in therapeutic antibodies due to shear in the presence of a solid–liquid interface. Therefore, filling systems and processes should be well understood and carefully selected for each drug product, especially for any protein molecules that are known to be highly sensitive to shear stress.

Though the filling system is often a fixed parameter due to the equipment setup in the filling line in a given manufacturing facility, a well-designed, product-specific pump filling study and/or recirculation laboratory study is recommended to evaluate the molecule susceptibility to filling stress and to identify any potential risk associated with the filling process. This type of study can be used to optimize filling parameters and confirm that the product quality attributes are not compromised during a worst-case filling process. The key for the suggested laboratory study is to use a representative filling pump (filling mechanism, contact material) with representative filling parameters such as nozzle size (time-over-pressure filling technology), filling speed, filling needle size, *etc*. During the technology transfer to a clinical or commercial manufacturing facility, an engineering run is typically performed to confirm the process feasibility.

### Storage of Protein Drug Products

Achieving desirable storage stability is one of the major challenges in the development of protein drugs. Proteins can experience a variety of environmental stresses during storage and degrade *via* different pathways. Interfacial stress is one of the primary causes of protein instability during storage. Protein drug products can be stored in various container–closure systems, such as vials, prefilled syringes, and stainless steel containers, and during IV infusion, in disposable plastic bags. Depending on the container–closure systems used, proteins can be exposed to various types of interfaces during storage, such as air–liquid interface, solid–liquid interface, and oil–water interface. Understanding the mechanisms of interface-induced protein instability is of great significance to the development of stable protein formulations and selection of appropriate storage conditions.

#### Exposure of Drug Products to the Air–Liquid Interface During Storage

Aqueous solutions of therapeutic proteins are exposed to the air–liquid interface due to the vial headspace or when bubbles are present. Proteins can adsorb to the air–liquid interface and form a viscoelastic interfacial film (13), with the same mechanism as that described during stirring above. Agitation during shipping can enhance air–liquid interface–induced protein aggregation by increasing the area of air–liquid interface and by accelerating the transport of denatured proteins from the air–liquid interface back to the bulk solution. Liu *et al.* showed that compression of the air–liquid interface promotes aggregation at the interface and consequent release of aggregates/particles into the bulk solution (62).

Agitation studies are routinely carried out during formulation and process development to assess the air–liquid interfacial stability of therapeutic proteins. The variables tested in agitation studies usually include method of agitation (such as stirring and shaking), intensity of agitation, size of headspace (in vials) or bubble (in prefilled syringe), and container orientation. Adsorptive loss of protein, structural and conformational change, aggregation, and particle formation are usually the key parameters to monitor in an agitation stability study. A number of techniques can be used to monitor the adsorption of proteins to the air–liquid interface and the strength of protein–protein interaction at the interface, including confocal microscopy, interfacial ellipsometry and X-ray/neutron reflectivity, and dilatational interfacial rheology (63). Other techniques, such as Fourier transform infrared spectroscopy (FTIR) and nonlinear vibrational spectroscopy, can be used to probe the structure and conformation of proteins at the interfaces (63). Surfactants, such as polysorbates, can prevent the adsorption of proteins to the air–liquid interface and are very effective at mitigating air–liquid interface–induced protein instability. Formulation conditions that can help improve protein colloidal stability may also enhance air–liquid interfacial stability of proteins.

#### Exposure of Drug Products to the Solid–Liquid Interface During Storage

The solid–liquid interface of container–closure systems is another type of interface that proteins encounter during storage. It has been shown that proteins can adsorb to various types of solid surfaces, including glass, plastic, rubber, and metal. Adsorptive loss of proteins can significantly reduce the dose that can reach patients and is of a particular concern to therapeutic protein products stored at a low concentration. A clinically well-studied example in this regard is insulin. It has been demonstrated that insulin can adsorb to various types of containers such as glass vials, plastic vials, and infusion bottles, which can result in 20–60% reduction in the dose available to patients (64–66). Adsorption to solid surfaces may also promote protein unfolding and aggregation. Denatured proteins, once desorbed from solid surfaces, can form potential nucleation sites and trigger protein aggregation in the bulk solution. Particles shed from solid surfaces encountered during manufacturing and storage, such as glass and stainless steel, can also act as potential nuclei and induce protein aggregation in bulk solutions (67,68).

In addition to physical degradation, contact with solid surfaces can induce chemical degradation and protein cleavage. Redox-active metals, such as iron and copper, can catalyze the oxidation of proteins stored in stainless-steel vessels (69,70). Smith *et al.* showed that copper can catalyze the cleavage of antibody in the hinge region (71). During formulation development, an important part of the container compatibility evaluation is the solution depletion study, which is used to evaluate the adsorption of proteins to container surfaces. In this study, liquid-state proteins are incubated in the containers of interest for various amounts of time. The protein concentration is measured before and after the incubation to obtain the adsorption–time curve. Surface adsorption of proteins can often be mitigated by adjusting the pH and ionic strength or by the addition of suitable excipients, such as surfactants, depending on whether adsorption is predominantly driven by electrostatic or hydrophobic interactions. However, formulation strategies used to mitigate against an electrostatically driven interaction (*i.e.*, storage in a glass container) may not be effective in a situation where hydrophobicity is driving the interaction between the protein and the container. Alternatively, a container material/coating that has a low adsorption profile to the protein of interest should be selected.

#### Exposure of Drug Products to the Oil–Liquid Interface During Storage

A third type of interface that proteins can be exposed to during storage is the oil–water interface. Silicone oil is commonly used as lubricant or coating in prefilled syringes and other pharmaceutical containers. Protein aggregation induced by silicone oil can be a challenge in the development and commercialization of therapeutic protein products (72), though the use of surfactant can mitigate this aggregation. Proteins can adsorb to the hydrophobic silicone oil–water interface and form a monolayer, which may result in the perturbation of protein structure and conformation (8). Agitation can significantly enhance silicone-oil–induced protein aggregation, presumably *via* increasing the area of silicone oil–water interface and accelerating the transport of structurally perturbed proteins from the oil–water interface to bulk solution. Carpenter *et al.* showed that there is a synergistic effect between silicone oil and agitation in the stimulation of the aggregation of monoclonal antibodies. The stability of monoclonal antibodies in the presence of silicone oil is better correlated with their colloidal stability, rather than their conformational stability (73). Randolph *et al.* demonstrated that ionic strength can affect the structure and aggregation propensity of antibodies adsorbed to silicone-oil-water interfaces (74). The sensitivity of therapeutic proteins to silicone oils can be studied by a silicone oil spiking study during formulation development. Considering the synergistic effect between agitation and silicone oil in the stimulation of protein aggregation, agitation stress is often applied as part of silicone oil spiking studies to simulate actual storage or transportation conditions. As with the solid/liquid interface, the use of surfactant and optimization of solution pH and ionic strength are important strategies for mitigating silicone-induced adsorption and aggregation, and most commercial formulations for biologics that will be delivered in prefilled syringes contain a surfactant. In the studies cited above by Randolph and Carpenter, surfactant was shown to ameliorate protein–silicone oil adsorption and subsequent protein aggregation. During the development of therapeutic proteins that are highly prone to silicone oil–induced aggregation, silicone oil-free syringes or syringes with other coatings such as Teflon™ can be considered. Additionally, compared with standard silicone coating, a novel cross-linked silicone coating is shown to be able to reduce protein aggregation and particle formation (75).

## IN-USE STABILITY STUDY OF INJECTABLE BIOLOGICAL DRUG PRODUCTS

The integrity of biological drugs for administration in clinical settings is very important because it affects not only the efficacy of the drugs but also the safety of patients. Understanding postproduction handling risks is therefore critical to the development of a robust product (76). The purpose of in-use stability testing is to establish a period of time for the preparation and administration of biological drugs used in the clinical settings while retaining quality within accepted specification. At this time, biological drugs are delivered *via* intravenous injection of either neat drug product or product that has been diluted with vehicle to achieve specific dosages in a clinical setting, or subcutaneously by various devices that are often self-administered by the patient. For IV administration, the commercially available fluids 0.9% sodium chloride injection, USP (normal saline), or 5% dextrose in water (D5W) are widely used as diluents. The infusion solutions for administration encounter various materials throughout the preparation and administration period, including syringes, tubes, IV bags, and in-line filters. In certain cases, it has been reported that the IV bag materials have an impact on aggregate formation (4). Additionally, the diluted solution may contain particles due to the lower concentration of surfactant after dilution (77); however, infusion solutions containing certain levels of protein aggregates or particles are not suitable for IV administration because they may reduce potency or increase immunogenicity (78). In-line filters are therefore often used to prevent any formed particulate matter from reaching the patient; however, protein adsorption to this filter or to other surfaces encountered throughout administration can lead to the patient receiving less than the intended dose, especially for very low dose therapies. Thus, in-use stability should be evaluated as a part of formulation development to avoid unforeseen problems.

Due to the high variability in clinical study designs, no specific guidance is available on defining the scope of in-use stability studies. Knowledge of how a product is intended for use in clinical settings is necessary prior to conducting the in-use stability study. However, in-use stability studies typically evaluate the compatibility of drug products with the diluents normal saline or D5W, along with various syringes, closed system transfer devices, tubes, IV bags, transfer lines, and in-line filters. Various concentrations of diluted drug products and flow rates should also be qualified. After preparation of the dosing solution, initial samples should be collected, then the infusion solutions should be exposed to the appropriate room temperature/room light conditions representative of the clinical environment before infusion. Both initial and infused samples should be tested, and intermediate samples can be tested, as needed. Generally, the samples should be aged longer than expected clinical use time in order to ensure the stability of the product throughout the longest allowable preparation, storage, and administration period. Microbial testing is not required when the infusion samples are stored at room temperature up to a maximum of 4 h because the assumption is made that the infusion solutions prepared aseptically and contained in sterile bags will remain sterile for this period of time. The analytical tests performed on the samples usually include those to probe levels of soluble aggregates, particles, charge variants, and fragmentation, as well as protein concentration, appearance, and pH. In particular, the HIAC liquid particle counter is the instrument often used for subvisible particle detection following guidance of USP <787> Subvisible Particulate Matter in Therapeutic Protein Injection, using small volumes and designed specifically for biologics, or USP <788> Particulate Matter in Injections. As stated in USP <788>, pass/fail limits for HIAC particle counts for small volume parenteral products (less than 100 mL) are 6000 per container for particles larger than 10 μm and 600 per container for particles larger than 25 μm. For larger volumes (more than 100 mL), HIAC particle pass/fail limits are 12 per mL for particles above 10 μm and 2 particles per mL for particles above 25 μm. Performing an in-use stability study allows the development team to understand any risks around interfacial interaction of the protein with the clinical components, as well as to define a safe operating space for clinical studies.

Subcutaneous delivery of therapeutic proteins is a fast-growing field, which often requires large quantities of drug to be administered within a small volume. High protein concentration drug products are conventionally delivered with different types of syringes and needles. Recently, prefilled syringes, autoinjectors, or on-body delivery systems have been increasingly utilized in subcutaneous drug delivery. In-use stability studies should include the evaluation of interfacial impact from the surfaces of those injection devices and the approaches to use them during administration.

## ANALYTICAL METHODS TO ASSESS INTERFACE-MEDIATED PROTEIN AGGREGATION

Biologics subjected to interfacial stress can generate a diverse assortment of aggregated species ranging in size from dimers and other soluble aggregates, to subvisible or micrometer-sized particles, to particles in the hundreds of micrometers that are visible to the unaided eye. Due to this size diversity, no single analytical technique is capable of providing a comprehensive assessment of the variety of aggregates that can be generated by interfacial stress. The subsequent section presents a brief overview of the analytical tools useful for characterizing aggregated species, including techniques that assess secondary/tertiary/higher order structure, size, and morphology. An analytical and characterization strategy needs to be developed that utilizes techniques that provide insight into the composition, morphology, mechanism of formation, and quantitation (*e.g.*, mass, particle count) of the aggregated species. It is beyond the scope of this paper to delve into the intricacies and limitations of each technology, as such information has been thoroughly reviewed elsewhere (79–82). Instead, the reader is directed to these reviews and the various citations contained throughout to achieve the appropriate level of understanding.

### Analysis and Characterization of Soluble Aggregates

The quantitation and analysis of soluble aggregates (particularly dimers, but also higher molecular weight species up to tetramers) is an important element in assessing, characterizing, and mitigating interfacial stress-induced protein alterations. The analytical workhorse for simple quantitation of soluble aggregate content is size exclusion chromatography (SEC). The use of a multi-angle light scattering detector during analysis by SEC (SEC-MALS) can provide further information regarding the absolute molecular weight of the aggregate species. For situations where interprotein disulfide bonds may form due to the interfacial stress, capillary electrophoresis-sodium dodecyl sulfate (CE-SDS) can provide valuable insight, due to its convenient ability to be used in both reduced and nonreduced formats.

The classic spectroscopic techniques, including ultraviolet absorbance, circular dichroism (CD), infrared (FTIR), Raman, and fluorescence (intrinsic and extrinsic), are capable of measuring changes in secondary and/or tertiary structure of the monomeric or aggregated forms and may provide information that can yield a mechanistic understanding of the root cause of aggregation/particle formation. In hyphenated microscopy format, FTIR and Raman have found particular use in characterizing the nature of proteinaceous particles.

Light scattering techniques, such as static light scattering (SLS) and dynamic light scattering (DLS), can be useful for characterizing aggregates in the submicron range. The former, when used in combination with size exclusion chromatography (*e.g.*, SEC-MALS), is particularly convenient for the characterization of the absolute molecular weight of soluble aggregates. DLS can provide an assessment of the hydrodynamic radii and size distribution (although resolution is limited) of particles and is generally well suited to detecting small amounts of relatively large aggregates. Turbidimetry, which measures the reduction in light passing through a sample, and nephelometry, which measures the amount of forward scattered light produced by a sample, can both provide a coarse assessment of particle concentration. Differential scanning calorimetry may afford an understanding of the stability of the potentially altered conformational state of the perturbed native state or aggregated states. More specialized techniques, such as analytical ultracentrifugation (AUC), asymmetric flow field-flow fraction (AF4), and hydrogen deuterium exchange monitored by mass spectrometry (HDX-MS) or FTIR (HDX-FTIR), may also be used to characterize soluble aggregates formed due to interfacial stress.

A convenient way to summarize the various measurement approaches describe above is by categorizing them according to what aspect of the aggregation process they are measuring (83):

1. Changes in protein secondary and tertiary structure
  - Circular dichroism spectroscopy (CD)
  - Fourier transform infrared spectroscopy (FTIR)
  - Raman spectroscopy
  - Fluorescence spectroscopy
    i. Intrinsic
    ii. Hydrophobic extrinsic dye binding (Thioflavin T (Th T), Sypro Orange, 8-anilinonaphthalene-1-sulfonic acid (ANS), 4,4′-dianilino-1,1′binaphthyl-5,5′-disulfonic acid (bis-ANS))
  - UV absorbance spectroscopy
  - Differential scanning calorimetry (DSC)
  - Hydrogen–deuterium exchange monitored by mass spectrometry or FTIR (HDX-MS or HDX-FTIR)
2. Changes in the number of aggregates and particles and in the amount of monomeric protein
  - High/ultra performance liquid chromatography
  - Capillary electrophoresis-sodium dodecyl sulfate (CE-SDS)
  - Static light scattering (SLS)
  - Multi-angle light scattering (MALS)
  - Dynamic light scattering (DLS)
  - Analytical ultracentrifugation (AUC)
  - Asymmetrical flow field-flow fractionation (AF4)

### Analysis of Submicron and Subvisible Particles

Submicron particles are generally defined as those particles between 100 and 1000 nm in size. This size range will likely gain more attention as the U.S. Food and Drug Administration Guidance for Immunogenicity Assessment for Therapeutic Protein Products states that sponsors should strive to characterize particles in this smaller size range (84). At present, there are two primary techniques for characterizing particles in this size range: nanoparticle tracking analysis (NTA) and resonant mass measurement (RMM). NTA counts and sizes particles by tracking the Brownian motion of individual particles within the sample of interest. Tian *et al.* have recently contributed an evaluation of NTA and its application in characterizing submicron, proteinaceous particles (85). The RMM method (also referred to as SRM, or suspended microchannel resonator) relies on the measurement of a particle’s buoyant mass to calculate a variety of useful parameters, including particle size and concentration. Like flow imaging (described later), this method can be particularly useful in differentiating silicone oil droplets from proteinaceous aggregates due to natural differences in buoyancy (86). An industry effort to use these two methods to survey the amount of submicron particles in marketed product is underway and the results should be available soon.

Subvisible particles (SbVP) are typically classified as those particles in the range of 1 and 100 μm. Particles in this range must be assessed as part of batch release and are also an important part of comparability testing and process development. The batch release methods, light obscuration (LO) or membrane microscopy analysis, for these particles/protein aggregates are described in several pharmacopeia chapters (33,87–89). The LO method is an optical method in which the amount of light passing through the aliquot being tested is measured. Whenever a shadow passes over the detector due to obscuration of the light, this is assumed to be a particle; the size is determined from the 2D shadow, and each individual shadow counted. For the membrane microscopy method, the sample is filtered, and then the particle size and number are determined by manual analysis of the filter (33,87–89).

### Characterization of Particle Morphology

As the inherent heterogeneity of proteinaceous particles became better understood, it became apparent that the compendial methods were not sufficient for characterization and root cause analysis (59,60). As a result, new method development was needed to provide a more detailed description of the particles formed during process development and characterization. The particles should be assessed for size, number, morphology, chemical modifications, and conformation, if they are proteinaceous (90,91). Many review articles describe the different techniques available for characterization (80,81,92), with the compendial chapter <1787> (33) providing an overview of the methods and their strengths and weaknesses. Many of these are used for analysis of monomers and particles across all sizes, including the biophysical methods described previously.

Particle morphology can be detected using flow imaging (FI). In this technique, optical images are captured as the sample flows through the detector and are subsequently analyzed. In addition to size and number, the images can be analyzed for brightness, shape, and Feret diameter. This method can help differentiate protein particles from fibers, solid spheres, or silicone oil droplets. FI is useful for root cause analysis; however, the method is not as robust as LO, and at present, there is no linkage of FI particle counts to historical specifications using LO as the test methodology. Thus, while flow imaging is a powerful characterization technique, it is not currently a batch release method with the robustness necessary for routine application in a GMP or quality facility. Both LO and FI are optical methods and, thus, depend on differences in refractive index between particle and surrounding solution. The National Institute of Standards and Technology (NIST) has done extensive studies comparing these methods to understand why the size distributions obtained from the same sample are different, and a thorough discussion of the confounding issues has been published (93).

USP Chapter <1> requires parenteral dosage forms to be essentially free from visible particulate matter (94). While the precise phrase “essentially free” may not appear in the analogous chapters of other global pharmacopeia, the underlying intent of such chapters is the same. Visible particles are most readily assessed by adhering to the basic approach outlined in the three International Council for Harmonization (ICH) region’s pharmacopeia (89,95,96). Briefly, the pharmacopeia require inspection against black and white backgrounds using a light intensity of 2000–3750 lx from a white light source. Even in these controlled lighting conditions, the detection of visible particles is probabilistic and depends on many attributes, including both observer- and particle-specific characteristics. As such, the probability of detecting a single spherical particle only begins to approach 100% for particles that are ~ 200 μm and larger, while trained operators may be able to detect particles in the 50–100-μm range (97). If more enhanced visible particle detection capabilities are desired, one can consider the use of manual, table-top inspection machines such as those from Seidenader (V90-T or VPE) or Bosch Packaging Technology (APK series or ETAC ProView). In some instances, these machines can be configured with high-resolution cameras to aid in detection of visible particles.

### Tools for the Evaluation of Interfacial Rheology

The response that an interfacial film of protein and/or surfactant exhibits when a force is applied to the film provides a wealth of information about the nature of the molecules adsorbed to the interface. Interfacial rheology, which measures this relationship, is therefore a valuable tool to understand protein aggregation at interfaces. Fuller *et al.* wrote a detailed review about the rheology of complex fluid–fluid interfaces (98). A brief overview of some of the available techniques for measuring the interfacial rheology of protein/surfactant films is presented here.

Interfacial rheology is typically performed either as a shear measurement under constant surface area or as a dilatational measurement where the surface area is uniformly compressed or expanded. Interfacial shear measurements performed with traditional rotational rheometers, which are typically employed for bulk rheological measurements, are the most common due to the availability of the instruments. A bicone disk geometry allows for the measurement of interfacial viscoelasticity of protein films (99) but can suffer from lack of sensitivity due to bulk drag on the geometry. The double-wall ring geometry improves on sensitivity by reducing bulk drag and has also been successfully employed for the measurement of protein films (100). However, since the viscoelasticity of interfacial films can be very low, traditional rotational rheometers do not always offer sufficient sensitivity.

Academic labs have developed a variety of dedicated interfacial rheological methods, and some of these are now commercially available. An interfacial shear rheometer based on a thin magnetic rod floated at the fluid–fluid interface (101) provides good sensitivity for the measurement of interfacial protein films (102) and is commercially available. To further increase the sensitivity of interfacial shear measurements, several active microrheology measurements that use smaller probes can be employed, thus increasing sensitivity to the interfacial film rather than the bulk fluid(s). A microrheometer based on the rotation of magnetic 300 nm rods successfully measured the interfacial viscoelasticity of a protein film over four orders of magnitude as the film aged at the air–liquid interface (103). Another microrheometer based on the rotation of magnetic disks has been used to characterize the viscoelastic properties of lipid films, but could be readily adapted to measure interfacial protein films (104).

While interfacial shear rheometers are ubiquitous, dilatational measurements obtained after the surface area is uniformly compressed and/or expanded offer additional insight into the properties of protein films. Notably, the uniform compression of protein films adsorbed to an air–liquid interface highlighted the difference in aggregation propensities of two mAbs (105), as well as showed that particle shedding from the interface depended on the extent of compression and the length of exposure of the antibody to the air–liquid interface (13).

### Tools for the Quantification of Adsorption Dynamics

As discussed throughout this paper, the adsorption of therapeutic proteins to interfaces can drive the lateral aggregation of those confined proteins. Therefore, measuring both the adsorption dynamics of proteins and the interfacial phase behavior can play an important role in biotherapeutic development. In parallel, the role of surfactants in multicomponent systems with proteins can be quantified, as the competitive adsorption process is known to limit protein adsorption.

One of the oldest and most ubiquitous methods for measuring adsorption to fluid–fluid interfaces is surface (interfacial) tension. Surface tension was used to predict the aggregation propensity of a panel of mAbs and, therefore, could serve as a method for identifying problematic protein molecules early during development (10). Two common techniques are available commercially for the measurement of quasi-static surface tension on timescales of seconds to hours: the Wilhelmy plate method and the pendant drop method. The Wilhelmy plate method directly measures the force acting on a flag placed at the fluid–fluid interface. This method typically requires milliliters of solution to measure. The pendant drop (or pendant bubble) apparatus works by allowing a ~ 1-mm-diameter drop of fluid to equilibrate in another bulk fluid, allowing for protein and surfactant to adsorb to the interface. The pendant shape of the drop is a balance between the tension of the interface and gravity acting on the drop, as described by the Young–Laplace equation. The pendant drop technique has been used to examine the kinetics of protein adsorption to fluid–fluid interfaces (106–108). This method typically requires ~ 5 mL of solution to measure the dynamics of surface tension; however, there have been recent developments to build a microfluidic-based pendant bubble/drop apparatus that requires ~ 20 μL of solution (109,110).

As proteins begin to populate the interface from the aqueous buffer, the surface concentration increases and lateral interactions between proteins will define a 2D phase behavior that can be visualized using microscopy tools such as Brewster angle microscopy (BAM) or fluorescence-based microscopy. The execution of a BAM experiment mirrors the Wilhelmy plate setup described above, where proteins adsorb to the air–water interface as a function of time. BAM images *in situ* changes in refractive index at the air–water interface. At 53°, the Brewster angle of an air–water interface, minute changes in refractive index at the interface can be imaged, visualizing aggregation as well as multilayer formation (111,112).

Fluorescence-based microscopy techniques can also quantify adsorption and examine protein aggregation at various interfaces. These techniques typically require an extrinsic fluorophore to label the protein or molecule of interest, but carefully choosing the dye and limiting the labeling can allow for excellent visualization while not compromising the interfacial behavior of the molecule. Confocal microscopy, which provides good axial resolution for selectively observing interfacial phenomena, can be used to quantify the amount of protein adsorbed to an interface either in a Langmuir trough (113) or in a microtrough requiring less than 100 μL of solution (10). Total internal reflection fluorescence (TIRF) microscopy utilizes an evanescent wave to selectively visualize molecules within a few hundred nanometers from a solid–liquid or liquid–liquid interface. TIRF has been used to track single protein molecules at an oil–water interface to determine adsorption and aggregation kinetics (114).

The change in surface tension upon changing the surface area available to an adsorbed film of protein and surfactant also provides useful information about adsorption dynamics. A Langmuir trough provides a controlled compression/expansion rate and allows for the simultaneous measurement of surface tension and other properties (6,14). The decrease in surface tension upon compression suggested strong protein–protein interactions in adsorbed films of antibodies (6,14), and the hysteresis experienced upon compression and then expansion may indicate loss of material from the interface into the bulk and/or an irreversible change in the protein film due to compression. Protein films formed at the air–liquid interface can then also be transferred to a flat, solid substrate for subsequent topological characterization with atomic force microscopy, a combination that was used to better understand particle formation of an antibody at the air–liquid interface (14).

A multitude of label-free methods can be used to measure the adsorption of proteins and surfactants to solid–liquid and liquid–liquid interface. Quartz crystal microbalance with dissipation monitoring (QCM-D) measures the change in resonant frequency of a quartz crystal as different materials adsorb to the surface. The crystal may be functionalized with various materials like silicone oil, polymers, and metals. QCM-D measures the wet mass, which includes the molecules of interest as well as any hydration layer around those molecules, and has been used to study therapeutic protein adsorption to silicone oil–water interfaces (115). While QCM-D can provide sensitive equilibrium adsorption measurements and can also measure the viscoelasticity of the adsorbed films, it is not well suited for measuring adsorption kinetics. However, several other label-free techniques provide highly sensitive adsorption kinetics at solid–liquid and liquid–liquid interfaces. Well-established tools to examine the rate of adsorption and the developing thickness of the protein-based film include ellipsometry (116,117) and surface enhanced Raman spectroscopy (118,119). Recently developed tools include optical waveguide lightmode spectroscopy (OWLS), surface plasmon resonance (SPR), biolayer interferometry (BLI), and sum frequency generation (SFG).

OWLS and SPR work on a similar principle of producing an evanescent wave to measure the local change in refractive index within approximately 100 nm of the surface due to adsorption of molecules. Both have been used to understand the interplay between surfactants and proteins adsorbing to solid–liquid interfaces (120,121). However, due to the sensitivity to refractive index changes, OWLS and SPR can both suffer from matrix effects. In contrast, BLI measures a wavelength shift due to the thickness and optical properties of an adsorbed layer to a solid substrate, so it can therefore be used in complex sample matrices. The substrate can be modified, as with the other techniques mentioned above, and this tool has been used to measure protein adsorption and desorption kinetics to various polymer-modified surfaces (122). SFG uses two incident beams, one in the visible and one in the IR to create a “sum frequency.” The IR beam is broadly tuned to match the vibrational transition of the C–H or amide I group. SFG can only probe systems where inversion symmetry is broken, which occurs at the interface and not in the bulk. Because of the interfacial sensitivity, SFG can be used to simultaneously quantify adsorption rates, secondary structure, and structural orientation at the interface (123).

Due to the advantages and disadvantages of each of these methods for measuring protein and surfactant adsorption dynamics, a combination of techniques is likely necessary to provide a mechanistic understanding of protein adsorption and response to stress at the interface.

## RECOMMENDATIONS

Biologic molecules encounter multiple types of interfaces during development, manufacturing, and throughout the lifetime of the product. Here, we have highlighted many of the interfacial stress conditions to which proteins are exposed, as well as the effect of these stresses on the stability of the drug substance and drug product, and methods used to characterize. While some mitigation strategies, such as the addition of surfactant, are relatively common, other risk assessment tools and strategies around evaluation of interfacial sensitivity of a given protein should be considered. Each protein has its own unique sensitivities, and in order to develop a robust drug product, it is important to understand the different risks for each molecule. While some recommendations have been made within each section, here we provide a summary of recommendations during different stages of biologics product development.

### Drug Substance

When working with an inherently sensitive molecule, development and manufacturing of a drug substance may be of particular concern, as during the drug substance manufacturing process for many molecules, no surfactant is present until the final step. Until the risk of exposure to interfacial stress has been evaluated, a conservative operating space should be employed and exposure to excessive interfacial stress should be avoided. Experience is gained as each molecule moves through the development process, but risk assessment tools should be applied to better understand the effect of interfacial stress on a given protein. In particular, agitation studies and freeze/thaw studies are often performed early in the development process. In these studies, the product is exposed to agitation stress or to multiple freezing and thawing cycles, respectively, then characterized *via* size exclusion chromatography and particulate analysis, as well as any specific stability indicating assays important for the molecule of interest. For example, an agitation study may include agitation of the molecule on an orbital shaker at moderate rate for 1 to 7 days, or a shorter period of agitation *via* a wrist action shaker. During the early development process, an average of five freezing and thawing cycles is often performed, where the molecule is cycled between − 70°C and room temperature, and then analyzed for HMWS and particle formation after each cycle to look for any upward trends. While each company has their own operating space and product specifications, any significant increase in either soluble aggregates or particle counts with exposure to interfacial stress should be examined further and potential root causes should be evaluated. These early screening tools can provide insight into the stability of the product under stress conditions and can allow the development team a design space in which to operate while designing the manufacturing process.

### Drug Product

Each drug product should also be evaluated for sensitivity to different interfaces during manufacturing, storage, and transportation. During drug product manufacturing, the protein is exposed to freezing/thawing; shear stress and stress at the air/water interface during stirring, pumping, and filling operations; and solid/liquid interfacial stress during filtration and with exposure to different manufacturing components. During transportation and storage, both container compatibility and ruggedness with respect to agitation are critical for the long-term stability of a molecule. Thus, sensitivity to freezing and thawing, shear and air/water interfacial stress, and solid/liquid interfacial stresses should all be examined. Because degradation of surfactant on stability can occur, studies on aged drug product and/or studies using surfactant levels representative of the end of shelf life should be performed.

First, in addition to the freezing and thawing studies that are often performed on small volumes early in development, scale down models are often used to evaluate performance with freezing and thawing in smaller containers representative of the surface area to volume ratio of the target storage container for commercial drug substance manufacture. In addition to the agitation studies performed above, after a drug product presentation is locked, more specific agitation studies should be performed to ensure the product is robust enough to handle the interfacial stress sustained during transportation. Performing these studies at the target fill volume provides representative conditions for both the air/water interface and the container compatibility. In particular, shock testing and shipping tests are often performed on representative drug product vials prior to commercialization. In these shipping studies, the drug product is shipped and exposed to real-world conditions that may arise during transportation. In particular, using aged drug product for these studies can account for any surfactant degradation that may occur over time in certain products, demonstrating the robustness of the formulation to protect against transportation-induced interfacial stress, even at the end of the use period.

Container and manufacturing component compatibility is also critical. While the majority of commonly used containers and manufacturing components can be considered low risk for causing substantial stability challenges, it is still important to perform a risk assessment and/or a study to ensure no surprises emerge on stability. For example, small volumes of bulk drug product can be incubated in the container(s) of choice for 1 to 3 months under recommended storage conditions and under appropriate accelerated conditions for the protein of interest. Similarly, small pieces of each component of a given manufacturing scheme can be incubated with the bulk drug product for 3 to 7 days at the temperature anticipated for manufacturing, then compared to protein simply held at the same temperature in an adjacent vial. Filtration is likely the unit operation where a solid/liquid interface problem will emerge; most importantly, protein and surfactant concentration should be measured before and after filtration to ensure no material has been lost to the filter. If material is lost in the first volume to pass through the filter (for example, in the first 100 mL of a clinical manufacturing scale filter), but a saturation point is reached, a surge vessel can be used. Alternatively, a filter flush volume can be built into the manufacturing instructions in order to saturate the filter and allow the remainder of the material to pass through without loss of protein concentration. Different filter types can also be explored, as some may be more likely to cause protein adherence than others.

In addition to agitation and interfacial screening tools, shear rates for different mixing and filling parameters can be modeled, and mini-piloting tools can be utilized to expose the molecule to these levels of shear. Interestingly, in some cases, problems do not manifest in immediate aggregation, but aggregates may form over time in solutions that have been exposed to stress. Thus, it is also recommended to expose the molecule to the anticipated stress condition and then set it on stability in order to observe any lasting effects the exposure to interfacial stress may cause. Aggregates and particle counts should be measured before stress, immediately after stress, and again after storage at recommended and accelerated storage conditions.

The described risk assessment tools can also be used to set an appropriate level of surfactant to be included in a given formulation. For example, formulations with different levels of surfactant can be exposed to the same stress conditions, then evaluated for formation of particles and soluble aggregates. Surfactant degradation should also be considered; in some cases, surfactants can degrade from either oxidation or enzymatic activity. Degradation kinetics should be well-understood to enable prediction of the remaining surfactant levels at the end of shelf life. Subsequently, enough surfactant should be added to the formulation to ensure that enough intact surfactant remains at the end of the use period, so that even drug shipped to a site and administered to a patient in the last month of the use period will be stable and safe for clinical use.

### In-Use Stability

Once the drug product has been manufactured and has reached the clinical site, the steps used in product administration can still expose the molecule to stress conditions. A thorough description of in-use stability studies can be found in the “IN-USE STABILITY STUDY OF INJECTABLE BIOLOGICAL DRUG PRODUCTS” section above. For IV infusion, it is important to assess the stability of the product in the diluents recommended for the product’s administration. Because dilution of the drug product with the vehicle inherently dilutes the surfactant, as well, particulate formation can reemerge as a problem for sensitive molecules. In-use stability studies are therefore a critical part of the development process. These studies evaluate the stability of the molecule when exposed to different diluents and components used during product administration, including IV bags, IV sets, and in-line filters. If the product is to be administered subcutaneously, the stability of the product in the presence of the silicone oil and in contact with the administration device should be considered. Advances in patient drug delivery and increased interest in patient convenience have led to an increase in the use of autoinjectors and other self-administration devices. If these devices are to be used, the contact surfaces and solid/liquid interfaces encountered during use should be evaluated for their impact on the stability of the protein itself. In all cases, the infusion product should be tested for formation of high molecular weight species and particulate matter, as well as tested *via* any specific stability-indicating assay for the molecule of interest.

## CONCLUSIONS

Exposure of proteins to interfaces during development, manufacturing, and storage is inevitable. Each protein has its own unique sensitivities, and in order to develop a robust drug product, it is important to understand the different risks for each molecule. While some mitigation strategies, such as the addition of surfactant, are relatively common, other risk assessment tools and strategies around the evaluation of interfacial sensitivity of a given protein should be considered. In this commentary, we have described the interfacial conditions to which proteins are exposed, the effects interfacial exposure have on the stability of the protein, potential stability studies and risk assessment tools to understand the impact on each individual molecule, as well as new analytical techniques that are available to explore the behavior of different molecules at the air/liquid and solid/liquid interface. Understanding the particular sensitivities of a molecule throughout the development process is key to development of a robust drug product.
